# Compact filtering monopole patch antenna with dual-band rejection

**DOI:** 10.1186/s40064-016-2597-3

**Published:** 2016-06-24

**Authors:** Sun-Woong Kim, Dong-You Choi

**Affiliations:** Department of Information and Communication Engineering, Chosun University, Gwangju, Republic of Korea

**Keywords:** UWB, Dual-band rejection, Monopole patch antenna, WLAN, WiMAX

## Abstract

In this paper, a compact ultra-wideband patch antenna with dual-band rejection is proposed. The proposed antenna filters 3.3–3.8 GHz WiMAX and 5.15–5.85 GHz WLAN by respectively rejecting these bands through a C-shaped slit and a λ_g_/4 resonator. The λ_g_/4 resonator is positioned as a pair, centered around the microstrip line, and a C-type slit is inserted into an elliptical patch. The impedance bandwidth of the proposed antenna is 2.9–9.3 GHz, which satisfies the bandwidth for ultra-wideband communication systems. Further, the proposed antenna provides dual-band rejection at two bands: 3.2–3.85 and 4.7–6.03 GHz. The radiation pattern of the antenna is omnidirectional, and antenna gain is maintained constantly while showing −8.4 and −1.5 dBi at the two rejected bands, respectively.

## Introduction

The unlicensed use of ultra-wideband (UWB) set by the United States Federal Communications Commission requires the satisfaction of −41.3 dBm/MHz noise strength at a frequency band ranging between 3.1 and 10.6 GHz, along with 25 % fractional bandwidth and at least 500 MHz frequency bandwidth (Dullaert and Rogier 2010).

For UWB antennas, proposals have been developed to reduce interference from other UWB bands and for realizing a wide bandwidth with a stable radiation pattern (Kim and Min 2009; Kim and Kim 2010). To satisfy these requirements, a variety of structures for UWB antennas have been proposed, such as bow-tie antennas (Kiminami and Hirata 2004; Dadgarpour et al. 2009) that are easy to mount inside systems, elliptical antennas (Jang and Hwang 2008), Vivaldi antennas (Hood et al. 2008), and fractal antennas (Oraizi and Hedayati 2011).

Two bands coexist in the unlicensed use of UWB: IEEE 802.16 WiMAX (3.3–3.8 GHz) and IEEE 802.1a WLAN (5.15–5.85 GHz). However, these two bands are known to degrade the performance as a result of their interference with UWB communication systems.

Thus, this paper proposes the insertion of a λ_g_/4 resonator and a C-shaped slit into an antenna in order to reject both WiMAX and WLAN bands. To reject WiMAX, a pair of λ_g_/4 resonators is centered on the microstrip line, and a C-shaped slit is inserted into an elliptical patch. The proposed antenna satisfied the required bandwidth for UWB communication systems specified by the Federal Communications Commission, while maintaining dual-band rejection to prevent interference between bands.

The remainder of the paper is organized as follows: the “Background” section briefly introduces the proposal and design of the tapered slot antenna. The section, “Methodology and analyses of experimental data,” deals with the characteristics of the antenna, which were analyzed through a simulation and measurement process. The “Result and discussion” section presents the comprehensive results for the proposed antenna. Finally, in the “Conclusion”, we draw conclusions regarding the proposed antenna.

## Background

### Antenna design

For the structure of the proposed antenna, a λ_g_/4 resonator and a C-shaped slit were inserted into an antenna. The antenna was designed with an elliptical patch structure, in order to reject both WiMAX and WLAN. It was fabricated using the Taconic TRF-45 substrate, which is 1.62 mm in thickness and offers a relative permittivity of 4.5 and a loss tangent of 0.0035. The structure of the antenna is compact, with a total size is 40 × 35 mm^2^.

**Fig. 1:**
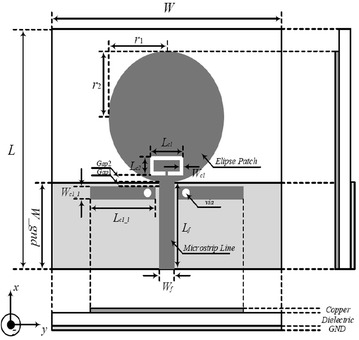
Structure of the proposed UWB monopole antenna with dual-band rejection

The design and analysis of the antenna were facilitated with HFSS, a commercial simulator tool available from Ansys. Its structure and design parameters are shown in Fig. 1 and Table 1 (Weng et al. 2010a, b).

**Table 1:** Parameters of the proposed antenna (mm)

L	35	W	40
L_f_	13.05	W_f_	2
r_1_	12	r_2_	10
L_c1_	6.8	W_c1_	3.2
L_c2_	3.2	L_c1_1_	11.6
W_c1_1_	2	W__gnd_	12
Gap1	0.6	Gap2	7.5

Two bands coexist for unlicensed use in the UWB:WiMAX (3.3–3.8 GHz) and WLAN (5.15–5.85 GHz). The proposed antenna rejects both of these bands using a λ_g_/4 resonator and a C-shaped slit (Sarkar et al. 2014; Wu et al. 2013).

**Fig. 2:**
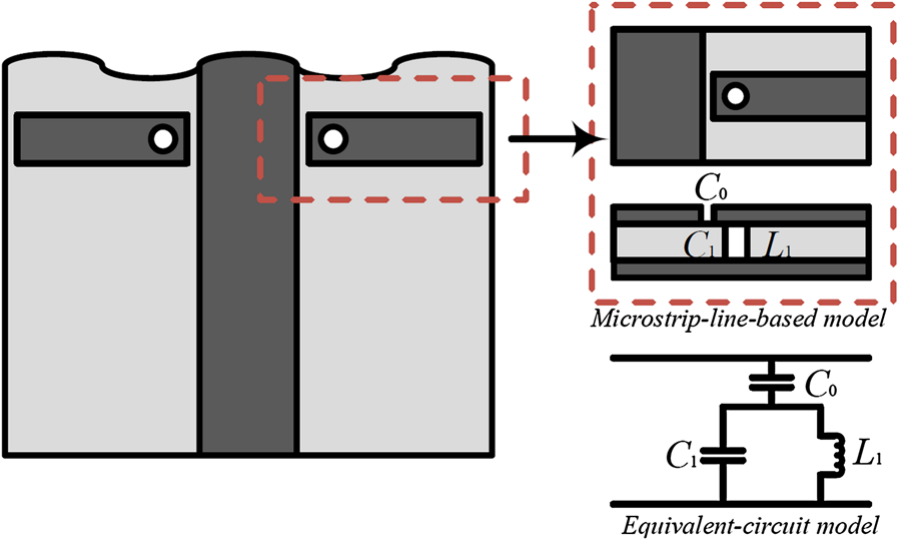
The mechanism of the proposed λ_g_/4 resonator

The equivalent circuit for the proposed λ_g_/4 resonator is shown in Fig. 2.

The capacitance C_0_ is the coupling generated between the microstrip line and the λ_g_/4 resonator. The capacitance C_1_ is derived from the voltage gradient between the λ_g_/4 resonator and the ground plane, whereas inductance L_1_ is generated due to the current flowing through the pin. The rejection frequency is derived as follows (Trinh-Van and Dao-Ngoc 2011).

1 $$f_{r} = \frac{1}{{2\pi \sqrt {L_{1} \left( {C_{0} + C_{1} } \right)} }}$$

In order to reject WiMAX, the following equation is used:

2 $$L_{c1 - 1} = \frac{{\lambda_{g} }}{4}$$

where L_c1_1_ is the length of the resonator, and λ_g_ is a guided wavelength.

3 $$\lambda_{g} = \frac{{\lambda_{0} }}{{\sqrt {\varepsilon_{eff} } }} = \frac{c}{{f_{r} \sqrt {\varepsilon_{eff} } }}$$

For the guided wavelength λ_g_, an effective dielectric constant ε_eff_ must first be determined, along with a suitable length for the microstrip line. Here, *f* denotes the frequency, and *c* denotes the speed of light in a free space. An effective dielectric constant ε_eff_ can be derived with the following equation:

4 $$\varepsilon_{eff} = \frac{{\varepsilon_{r} + 1}}{2} + \frac{{\varepsilon_{r} - 1}}{2}\left( {1 + \frac{12h}{w}} \right)^{ - 0.5}$$

where ε_r_ is the relative permittivity of the substrate, and *h* and *w* denote the substrate thickness and the width of the microstrip, respectively. Next, L_c1_ and L_c2_ are calculated such that the C-shaped slits reject WLAN, and these are derived as follows (Xu et al. 2012; Hong and Lancaster 2004):

5 $$L_{c1,c2} = \frac{{\lambda_{g} }}{2}$$

**Fig. 3:**
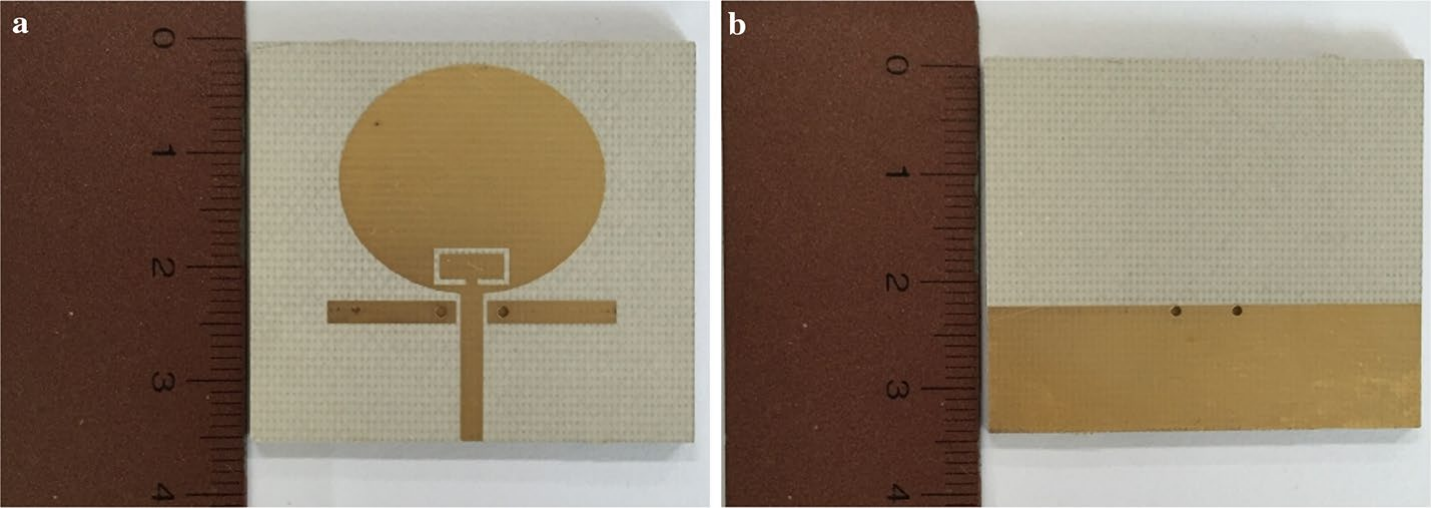
Size and look of the proposed antenna. **a** Front side, **b** Rear side

Figure 3 depicts the size and look of the proposed UWB monopole antenna with dual-band rejection.

## Methodology and analyses of proposed antenna

The antenna is expressed by the reflection coefficient Γ, which is the amount of reflected signal due to the impedance mismatch between the source and the antenna.

The VSWR is calculated as follows (Chang 2000):

6 $${\text{VSWR}} = \frac{{1 + \left|\Gamma \right|}}{{1 - \left|\Gamma \right|}}$$

When |Γ| = 0, optimal VSWR is obtained. This means that all power is transmitted to the antenna, and that there is no reflection. The impedance bandwidth of the antenna is defined at VSWR ≤ 2, and it is the reflected value of approximately 11 % input power (Chang 2000). Therefore, the proposed antenna has wide impedance bandwidth and powerful rejection-band characteristics.

**Fig. 4:**
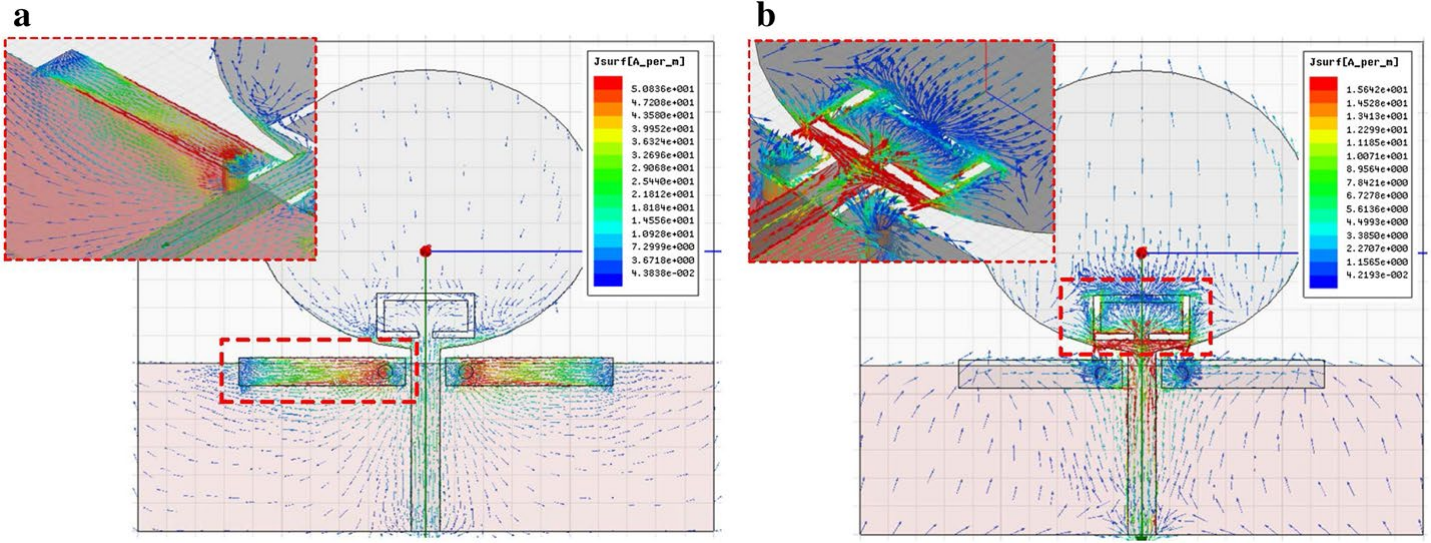
Simulated analysis of the current distribution for the proposed antenna. **a** 3.5 GHz band (WiMAX), **b** 5.5 GHz band (WLAN)

We analyzed the characteristics of the proposed antenna in terms of its ability to perform dual-band rejection by simulating its current distribution, as shown in Fig. 4.

**Fig. 5:**
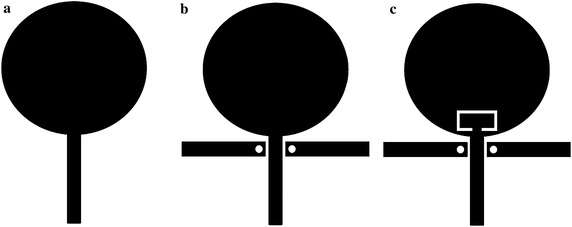
Three structuresfor the proposed antenna. **a** Structure-1, **b** Structure-2, **c** Structure-3

**Fig. 6:**
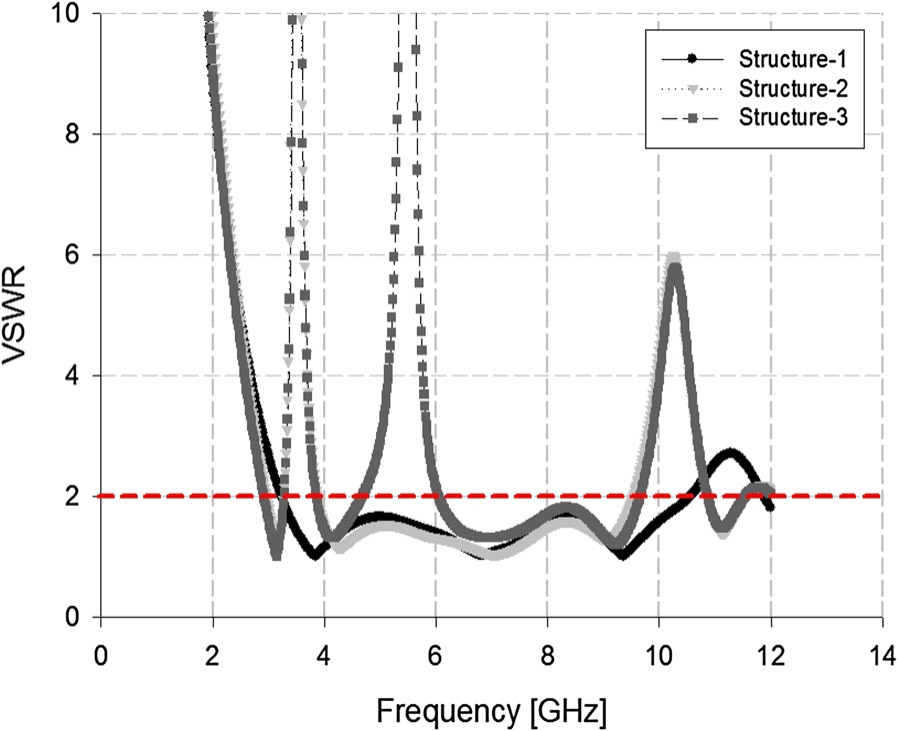
VSWR analysis using a simulationof the proposed antenna

Figure 4 shows the field distribution pattern on the patch, along with its modification with a C-shaped slit and λ_g_/4 resonators. The proposed antenna had a concentrated current at the λ_g_/4 resonator over the 3.5 GHz band (WiMAX), and a further concentration at the C-shape slits over the 5.5 GHz band (WLAN). The dual-band rejection and impedance bandwidth for the proposed antenna were analyzed for each structure using the voltage standing-wave ratio (VSWR). All three structures and their corresponding VSWR are shown in Figs. 5 and 6, respectively.

As shown in Fig. 6, Structure-1 converged with a VSWR ≤ 2 over the 3.27–10.48 GHz band, thereby satisfying the impedance bandwidth for standard UWB communication systems. Structure-2 successfully rejected the 3.27–3.87 GHz band, and its impedance bandwidth converged with a VSWR ≤ 2 at the 2.99–9.50 GHz band. Structure-3 successfully rejected both the 3.28–3.85 GHz band and the 4.7–6.03 GHz band, and its impedance bandwidth converged with a VSWR ≤ 2 at the 2.94–9.63 GHz band. Therefore, we demonstrated that dual-band rejection is feasible, and we verified that the proposed structure offers suitably high bandwidth for UWB communications.

**Fig. 7:**
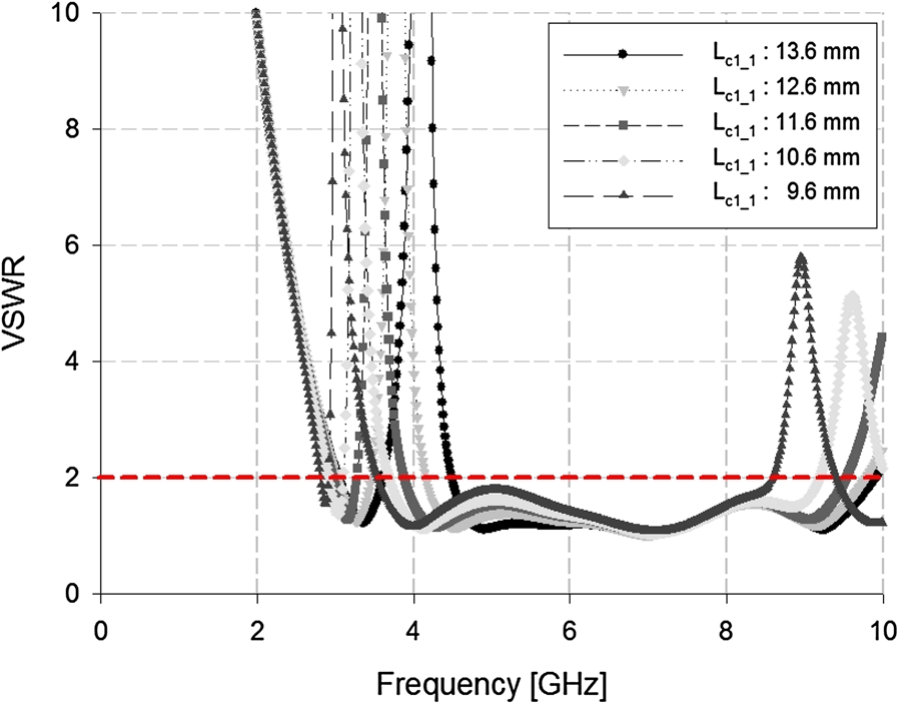
VSWR analysis of the λ_g_/4 resonator

Either the WLAN band or the WiMAX band can be rejected with physical changes to the C-shape slits or the λ_g_/4 resonator of the proposed antenna, respectively. Thus, the WiMAX band can be rejected through physical changes to the λ_g_/4 resonator, as shown in Fig. 7.

Figure 7 shows that, as the length of the λ_g_/4 resonator increased from 9.6 to 13.6 mm, the rejected band shifted to a higher frequency. At 11.6 mm, the WiMAX band was exclusively rejected.

**Fig. 8:**
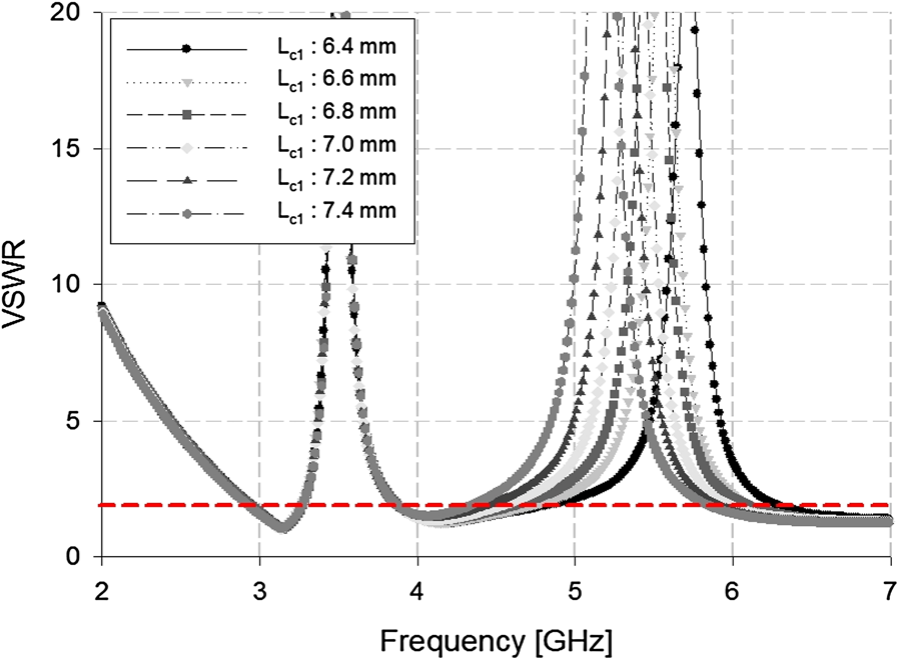
VSWR analysis of the C-shaped slit

The WLAN band can likewise be rejected by making physical changes to the C-shaped slits, as shown in Fig. 8.

Figure 8 shows that, as the length of the C-shaped slit increased from 6.4 to 7.4 mm, the rejected band shifted to a lower frequency. At 6.8 mm, the WLAN band was exclusively rejected.

**Fig. 9:**
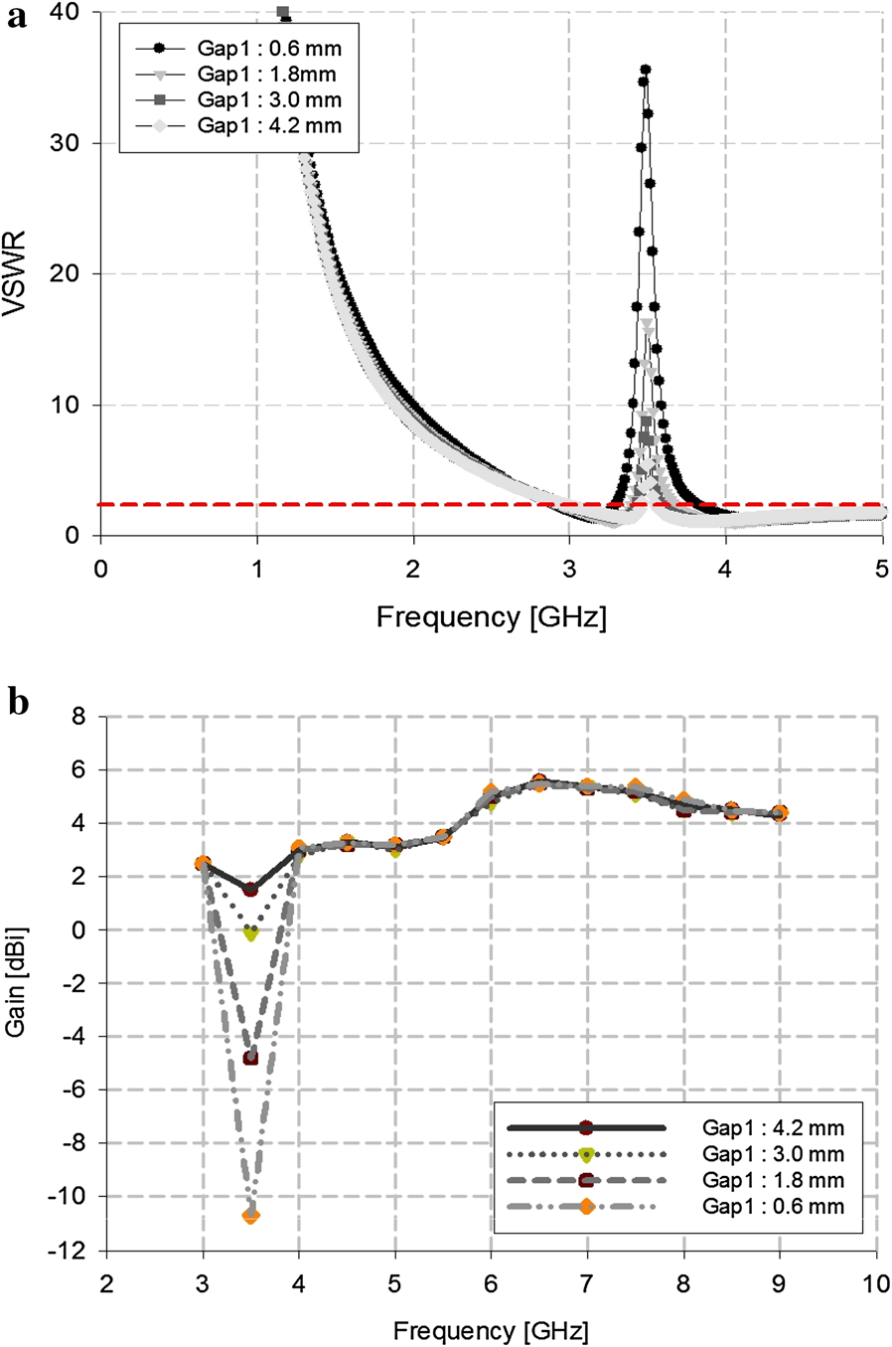
Analysis of the WiMAX-band rejection. **a** VSWR, **b** Antenna gain

In order to confirm the reliability of the band rejections with the proposed antenna, we analyzed the gaps between the antenna and the λ_g_/4 resonator and between the antenna and the C-shaped slit. Our analysis showed that a higher VSWR resulted in superior band rejections, owing to an impedance mismatch. We performed this analysis by varying the gap between the λ_g_/4 resonator and the antenna to 1.2 mm, the results for which (i.e., exclusively rejecting WiMAX) are shown in Fig. 9.

As shown in Fig. 9, as the gap narrowed between the antenna and the λ_g_/4 resonator, the VSWR increased and antenna gain reduced to below −10 dBi.

**Fig. 10:**
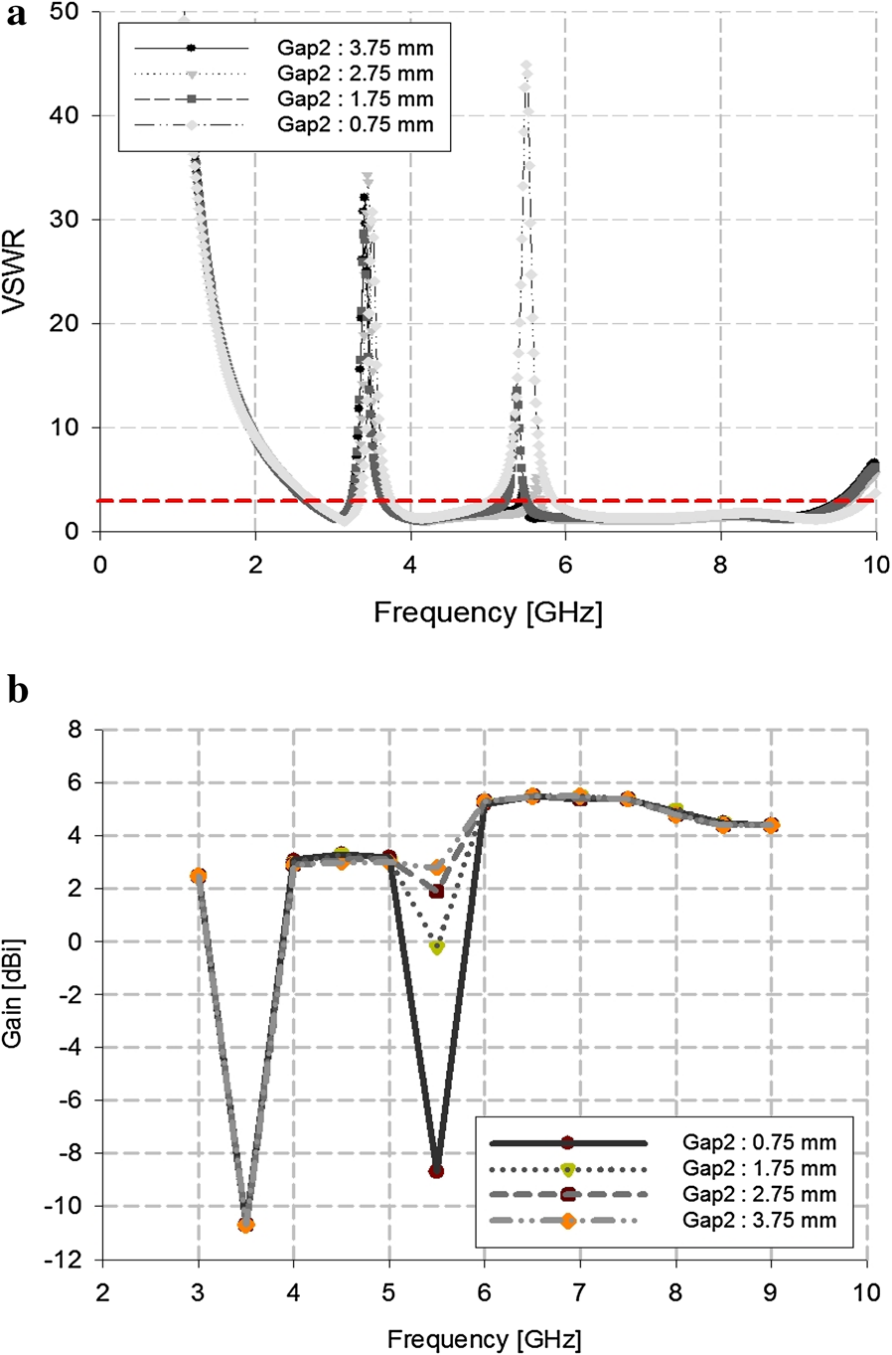
Analysis of the WLAN-band rejection. **a** VSWR, **b** antenna gain

We then modified the gap between the C-shaped slit and the antenna to 1 mm, and the results of this modification (i.e., exclusively rejecting WLAN) are shown in Fig. 10.

As shown in Fig. 10, as the gap narrowed between the antenna and the C-shaped slit, the VSWR increased and antenna gain reduced to below −8 dBi.

The overall characteristics of the proposed antenna in terms of its ability to reject WLAN and WiMAX are provided in Table 2.

**Table 2:** Analysis of the antenna’s band rejection

	VSWR	Gain (dB)
WiMAX		
Gap 1: 0.6 mm	37.5	−10.7
Gap 1: 1.8 mm	16.2	−4.8
Gap 1: 3.0 mm	8.6	−0.1
Gap 1: 4.2 mm	5.5	1.5
WLAN		
Gap 2: 0.75 mm	44.9	−8.7
Gap 2: 1.75 mm	13.5	−0.2
Gap 2: 2.75 mm	5.3	1.9
Gap 2: 3.75 mm	4.0	2.8

Table 2 shows that, for WiMAX, the antenna gain was −10.7 dB when Gap 1 was 0.6 mm. For WLAN, the antenna gain was −8.7 dB when Gap 2 was 0.75 mm. These results confirm the feasibility of the proposed antenna with dual-band rejection.

**Fig. 11:**
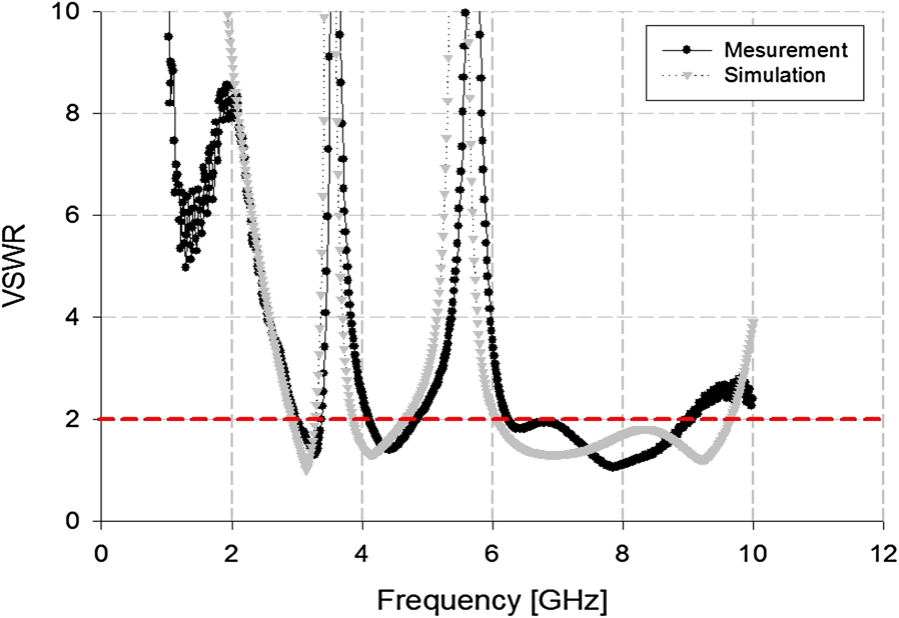
VSWR analysis of the proposed antenna

The UWB monopole patch antenna was also evaluated using a simulation, and these results are shown in Fig. 11.

As shown in Fig. 11, the impedance bandwidth of the proposed antenna satisfied dual-band rejection at two bands: 3.3–3.85 and 4.8–6.1 GHz. Its impedance bandwidth converged with a VSWR ≤ 2 over the 2.9–9.3 GHz band. Therefore, the simulation results are consistent with the measurement results.

**Fig. 12:**
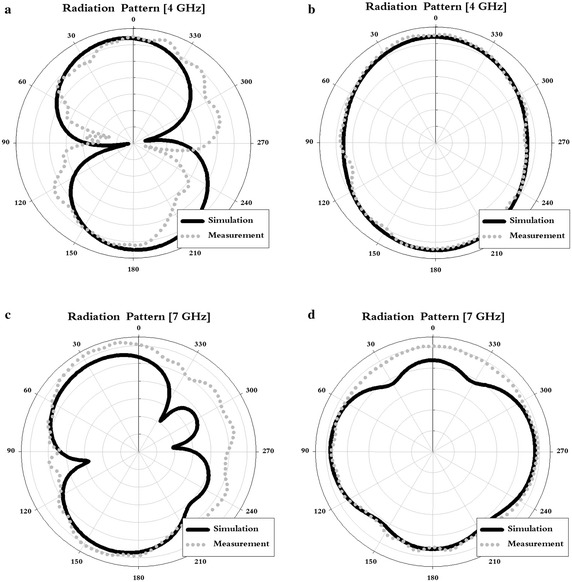
Analysis on the radiation pattern of the proposed antenna. **a** E-plane of 4 GHz (XZ-plane), **b** H-plane of 4 GHz (YZ-plane), **c** E-plane of 7 GHz (XZ-plane), **d** H-plane of 7 GHz (YZ-plane)

To further evaluate the proposed antenna, we used a simulation to analyze the radiation pattern along the E-plane (XZ-plane) and H-plane (YZ-plane) over two bands, 4 and 7 GHz, the results for which are shown in Fig. 12.

As shown in Fig. 12, the analysis of the radiation pattern of the proposed antenna demonstrated its omnidirectional characteristics at the 4 and 7 GHz bands.

**Fig. 13:**
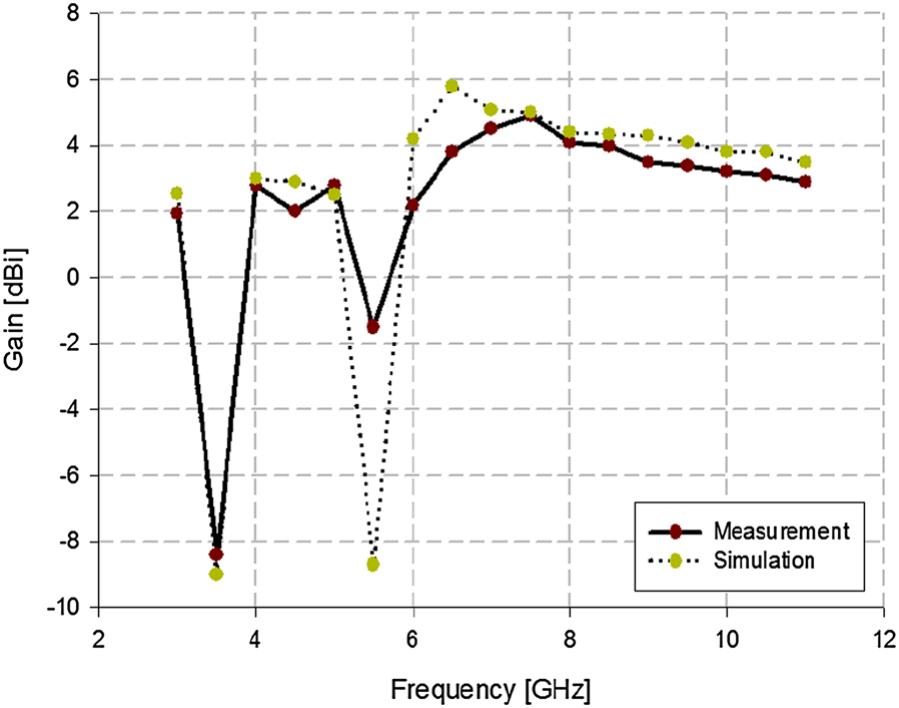
Analysis on the antenna gain of the proposed antenna

The antenna gain of the proposed antenna over all bands was also analyzed, and the results from this analysis are shown in Fig. 13.

As shown in Fig. 13, the analysis of the proposed antenna’s gain revealed that the simulation and measurement results were similar. However, the simulation analysis results and measurement results at 5.5 GHz were different by approximately 6 dBi. This was because of the loss in the physical size of the C-shaped slot during the production process. However, the band-rejection proceeded downward below 0 dBi, which is a suitable value.

## Results and discussion

The overall analysis results in Table 3 show that the proposed antenna offers the appropriate bandwidth for UWB communication systems, coupled with dual-band rejection to avoid interference between communication systems. These results also show that the radiation pattern of the antenna is omnidirectional.

**Table 3:** Overall analysis of the proposed antenna

Simulation results		
Impedance bandwidth	2.94–9.63 GHz	
Dual-band notched bandwidth	3.28–3.85 GHz	
	4.7–6.03 GHz	
Antenna gain [dBi]	3 GHz	2.53 dB
	3.5 GHz	−9.0 dB
	5 GHz	2.50 dB
	5.5 GHz	−8.7 dB
	7 GHz	5.09 dB
	9 GHz	4.31 dB
Measurement results		
Impedance bandwidth	2.9–9.3 GHz	
Dual-band notched bandwidth	3.3–3.85 GHz	
	4.8–6.1 GHz	
Antenna gain	3 GHz	1.95 dB
	3.5 GHz	−8.4 dB
	5 GHz	2.79 dB
	5.5 GHz	−1.5 dB
	7 GHz	4.51 dB
	9 GHz	3.50 dB

A mismatch was observed between the simulation results and the measured results for the proposed antenna. This occurred in two forms: the first pertained to errors during the manufacturing process, and the second to loss between the antenna and the connector. However, this mismatch is not a major problem with the proposed performance. On this basis, the impedance bandwidth was achieved with a VSWR ≤ 2, and the rejected band proceeded downward below 0 dBi.

The proposed antenna is compared to other antennas with rejected-band characteristics in Table 4. The advantage of the proposed antenna lies in its compact design and the fact that it has a dual-band rejection characteristic.

**Table 4:** Comparison of the proposed antenna and different antennas

Antenna	Rejected band (GHz)	Dimensions (mm^2^)
Trinh-Van and Dao-Ngoc (2011)	3.375–3.875	42.5 × 34
	5.325–6.150	
Dong et al. (2014)	4.96–5.42	38 × 44
	5.71–5.91	
Satyanarayana and Mulgi (2015)	3.2–4.2	38 × 50
Proposed antenna	3.2–3.85	40 × 35
	4.7–6.03	

## Conclusion

In this paper, we proposed a UWB monopole patch antenna with dual-band rejection. The impedance bandwidth of the proposed antenna satisfied VSWR ≤ 2 at the 2.9–9.3 GHz band and dual-band rejection from an impedance mismatch at the 3.3–3.85 and 4.8–6.1 GHz bands. Furthermore, we demonstrated that the antenna’s radiation pattern is omnidirectional, and that the antenna gain proceeded downward to below 0 dBi for dual-band rejection. Furthermore, the proposed antenna offers the advantage of dual-band rejection and a compact design, compared with different antennas.

The design of the proposed antenna was optimized through HFSS, a commercial electromagnetic simulator provided by Ansys. The antenna was designed using the Taconic TRF-45 substrate, which is 1.62 mm thick with a relative permittivity of 4.5 and a loss tangent of 0.0035.
